# Knockdown of CREB3L4 Inhibits Autophagy and Reduces Cisplatin Resistance in Gastric Cancer Cells by Downregulating BAG3

**DOI:** 10.1002/kjm2.70231

**Published:** 2026-05-07

**Authors:** Wen‐Ke Yin, Xue‐Mei Xie, Xiao‐Yan Song, Yue Xiang

**Affiliations:** ^1^ Department of Pathology Institute of Basic Medicine, North Sichuan Medical College Nanchong China; ^2^ Department of Pathology Affiliated Hospital of North Sichuan Medical College Nanchong China; ^3^ Lab of Ultrasonography Nanchong Oriental Hospital Nanchong China

**Keywords:** autophagy, Bcl‐2 associated immortality gene 3 (BAG3), cisplatin resistance, CREB3L4, gastric cancer

## Abstract

Cisplatin resistance remains a major obstacle in the treatment of gastric cancer (GC), and autophagy has been increasingly recognized as a key cytoprotective mechanism contributing to chemoresistance. CREB3L4 is an endoplasmic reticulum membrane‐bound transcription factor that has been shown to regulate the expression of Bcl‐2 associated athanogene 3 (BAG3), a co‐chaperone protein involved in autophagy and survival signaling in various cancers. However, whether the CREB3L4/BAG3 axis regulates autophagy and contributes to cisplatin resistance in gastric cancer cells remains unclear. In the present study, we investigated the roles of CREB3L4 and BAG3 in cisplatin‐resistant GC cell lines and found that CREB3L4 and BAG3 were overexpressed. Furthermore, BAG3 expression was inhibited following CREB3L4 knockdown in gastric cancer cells. Notably, knockdown of CREB3L4 inhibited autophagy and alleviated cisplatin resistance in gastric cancer cells by down‐regulating BAG3. In addition, silencing of CREB3L4 promoted apoptosis and inhibited cell proliferation, which was also associated with decreased BAG3 expression. Taken together, these findings indicate that depletion of CREB3L4 suppressed autophagy and reduced cisplatin resistance in GC cells by downregulating BAG3.

## 1 Introduction

Gastric cancer is currently the fourth most common malignancy worldwide and the third leading cause of cancer‐related mortality worldwide [1]. The burden of gastric cancer is particularly high in East Asia, especially in South Korea, Japan, and China, where both incidence and mortality remain markedly elevated, and it has become the second leading cause of cancer‐related death in China [2, 3]. At present, the overall treatment outcomes for gastric cancer remain unsatisfactory [4]. Recurrence and metastasis are the main causes of mortality in patients with gastric cancer, and the levels of cisplatin resistance‐associated proteins, including multidrug resistance‐associated protein 1 (MRP‐1) and P‐glycoprotein (P‐gp), have been reported to reflect the extent of chemoresistance [5]. Therefore, identifying the key molecular events associated with gastric cancer cell proliferation and metastasis remains essential for understanding its mechanisms of occurrence and progression.

CREB3L4 (also known as AIbZIP, Tisp40, or ATCE1) is an endoplasmic reticulum (ER) membrane‐bound transcription factor containing a basic leucine zipper (bZIP) domain [6, 7]. Previous studies have demonstrated that knockdown of CREB3L4 inhibits androgen‐dependent prostate cancer cell growth and induces G2/M phase arrest [7, 8, 9]. In addition, CREB3L4 has been reported to be overexpressed in hepatocellular carcinoma and gastric cancer and is involved in tumor progression through transcription induction of downstream target genes [10]. Moreover, it has been shown that CREB3L4 can induce Bcl‐2‐associated immortality gene 3 (BAG3), thereby influencing the progression of several types of cancers [9].

BAG3 protein is a member of the BAG family [11]. It has been shown to regulate several key biomarkers of cancer, such as cell survival as well as adhesion [12, 13]. Mechanistically, BAG3 not only interacts with Bcl‐2 to suppress apoptosis, but also affects the regulation of autophagy to promote the removal of misfolded and damaged proteins [14]. In addition, BAG3 has been implicated in the development of drug resistance in breast and ovarian cancers by mediating autophagy [15, 16]. However, whether CREB3L4‐mediated regulation of BAG3 contributes to autophagy and drug resistance in gastric cancer cells remains to be further studied.

In this study, we investigated the roles of CREB3L4 and BAG3 in cisplatin‐resistant gastric cancer cell lines. Our findings demonstrated that BAG3 is overexpressed in these cells and further revealed that CREB3L4 knockdown inhibits autophagy and reduces cisplatin.

## 2 Materials and Methods

### 2.1 Cell Culture and Treatment

MKN‐28 and AGS cells were bought from ATCC and cultured in DMEM supplemented with 10% fetal bovine serum (FBS; Gibco, Rockville, MD, USA) and 1% penicillin–streptomycin (Gibco, Rockville, MD, USA) in an incubator at 37°C with 5% CO_2_. Cisplatin‐resistant MKN‐28 and AGS cells were established following the method described in the study by Wang et al. [17]. Briefly, GC cells were incubated with cisplatin at a concentration of 0.1 μM for 2 weeks, then incubated with 1 μM cisplatin for an additional 2 weeks, and were then further maintained in 10 μM cisplatin. The cisplatin‐resistant MKN‐28 and AGS cells obtained after this stepwise induction were designated as MKN‐28/DDP cells and AGS/DDP cells, respectively. To assess the stability of cisplatin resistance, MKN‐28/DDP and AGS/DDP cells were cultured in cisplatin‐free medium for 4 weeks as withdrawal groups. Cell viability was then measured after treatment with various concentrations of cisplatin (0–128 μM) for 48 h, and the IC_50_ values were compared with those of cells continuously maintained in cisplatin‐containing medium.

To examine baseline differences in growth rate, parental and resistant cells were seeded into 96‐well plates at a density of 2 × 10^3^ cells per well. Cell viability was then determined by MTT assay every 24 h for five consecutive days, and the corresponding growth curves were generated.

### 2.2 Cell Viability Assays

MKN‐28, AGS, MKN‐28/DDP, and AGS/DDP cells were seeded into 96‐well plates at a density of 5 × 10^3^ cells per well. After 24 h, the cells were treated with various concentrations of cisplatin (0–128 μM) for 48 h. Subsequently, 20 μL of MTT solution (5 mg/mL; Promega, Madison, WI, USA) was added to each well and incubated for 4 h at 37°C. The resulting formazan crystals were then dissolved in 150 μL dimethyl sulfoxide (DMSO), and the absorbance was measured at 490 nm using a microplate reader (Bio‐Rad, Hercules, CA, USA). Cell viability was expressed as a percentage relative to untreated control cells, and IC_50_ values were calculated using GraphPad Prism 8.0.

### 2.3 Cell Transfection

CREB3L4 small interfering RNAs (siRNAs) were obtained from Riobio (Guangzhou, China). Cells were seeded into 6‐well plates and transfected with CREB3L4 siRNAs using Lipofectamine 3000 according to the manufacturer's protocol. The following siRNA sequences were used: siCREB3L4: 5′‐GGAUCUUCUUCGAGAUCAUTT‐3′ (sense), 5′‐AUGAUCUCGAAGAAGAUCCTT‐3′ (antisense); negative control (siNC): 5′‐UUCUCCGAACGUGUCACGUTT‐3′ (sense), 5′‐ACGUGACACGUUCGGAGAATT‐3′ (antisense). For overexpression experiments, the full‐length coding sequences of CREB3L4 and BAG3 were cloned into the pcDNA3.1(+) vector (Invitrogen, Carlsbad, CA, USA). Cells were then transfected with 2 μg of plasmid DNA using Lipofectamine 3000 according to the manufacturer's instructions. After 48 h of transfection, the cells were collected for subsequent experiments.

### 2.4 Colony Formation Assay

MKN‐28/DDP and AGS/DDP cells were seeded into 6‐well plates at a density of 500 cells per well and cultured for 14 days. Following the indicated treatments, the cells were fixed with 4% paraformaldehyde for 15 min, stained with 0.1% crystal violet for 20 min, and then photographed under a microscope (Olympus, Ishikawa, Japan). Colonies containing more than 50 cells were counted.

### 2.5 Flow Cytometry

MKN‐28/DDP and AGS/DDP cells were collected into 1.5 mL EP tubes, washed with cold PBS, and resuspended in 1× binding buffer. The cells were then incubated with FITC‐conjugated Annexin V and propidium iodide (PI) (BD Biosciences, San Jose, CA, USA) for 15 min at room temperature in the dark. Apoptosis was analyzed by flow cytometry (BD FACSCanto II, BD Biosciences), and the data were processed using FlowJo software (Tree Star, Ashland, OR, USA).

qPCR.

Total RNA was extracted from GC cells using TRIzol reagent (Invitrogen, Carlsbad, CA, USA). Quantitative PCR (qPCR) was then performed using the SYBR Ex Taq kit (Takara, Shiga, Japan) on a QuantStudio 3 system (Applied Biosystems, Foster City, CA, USA). The following primer pairs were used: CREB3L4 forward, 5′‐CTGCCCTGTCAAACCCTGTT‐3′ and reverse, 5′‐GCTTGTTACGGATTTTCCTCCT‐3′; BAG3 forward, 5′‐CAACAGCCGCACCACTAC‐3′; reverse, 5′‐CATTGGCAGAGGATGGAGTC‐3′; and GAPDH forward, 5′‐GGTCGTATTGGGCGCCTGGT‐3′ and reverse, 5′‐TACTCAGCGCCAGCATCGCC‐3′.

### 2.6 Western Blot

Cell lysates were extracted with RIPA buffer (Beyotime, Hangzhou, China). Proteins were separated by 10% SDS‐PAGE according to their molecular weight and subsequently transferred onto PVDF membranes. The membranes were then incubated with primary antibodies against CREB3L4 (1:500, Abcam, Cat# ab238127), BAG3 (1:1000, Abcam, Cat# ab47124), p62/SQSTM1 (1:1000, Abcam, Cat# ab109012), LC3B (1:2000, Abcam, Cat# ab192890), and GAPDH (1:3000, Santa Cruz, Cat# sc‐47,724) for 16 h at 4°C. After primary antibody incubation, the membranes were incubated with horseradish peroxidase (HRP)‐conjugated secondary antibodies (Beyotime, Jiangsu, China) for signal detection.

### 2.7 GFP‐LC3 Fluorescence Assay

To monitor autophagy activity, a GFP‐LC3 reporter construct was used instead of endogenous LC3 immunostaining. MKN‐28/DDP and AGS/DDP cells were transfected with the pEGFP‐LC3 plasmid using Lipofectamine 3000 (Invitrogen) according to the manufacturer's protocol. After 24–48 h of transfection, the cells were treated with cisplatin (10 μM) in the presence or absence of siCREB3L4 for 24 h. Subsequently, the cells were fixed with 4% paraformaldehyde for 15 min and washed three times with PBS. The nuclei were counterstained with DAPI (Beyotime, Cat# C1002) for 5 min. Fluorescence images were captured using a fluorescence microscope (Olympus IX73, Tokyo, Japan), and GFP‐LC3 puncta were quantified in at least 50 cells per group using ImageJ software to evaluate autophagosome formation.

### 2.8 Statistical Analysis

GraphPad Prism 8.0 software (GraphPad Software, San Diego, CA, USA) was used for statistical analysis. All data were initially assessed for normality using the Shapiro–Wilk test and for homogeneity of variance using Levene's test. Comparisons between two groups were conducted using Student's *t*‐test, whereas comparisons among multiple groups were performed using one‐way analysis of variance (ANOVA), followed by Tukey's post hoc test for multiple comparisons or Dunnett's post hoc test for comparisons against a control group, as appropriate. IC_50_ values were calculated by nonlinear regression using a sigmoidal dose–response curve fitting model ([Inhibitor] vs. normalized response—variable slope). Data are presented as the mean ± standard error of the mean (S.E.M.) from three independent experiments (*n* = 3), each performed with five technical replicates. A *p* value < 0.05 was considered statistically significant.

## 3 Results

### 3.1 CREB3L4 Was Highly Expressed in Cisplatin‐Resistant Gastric Cancer Cells

**FIGURE 1:**
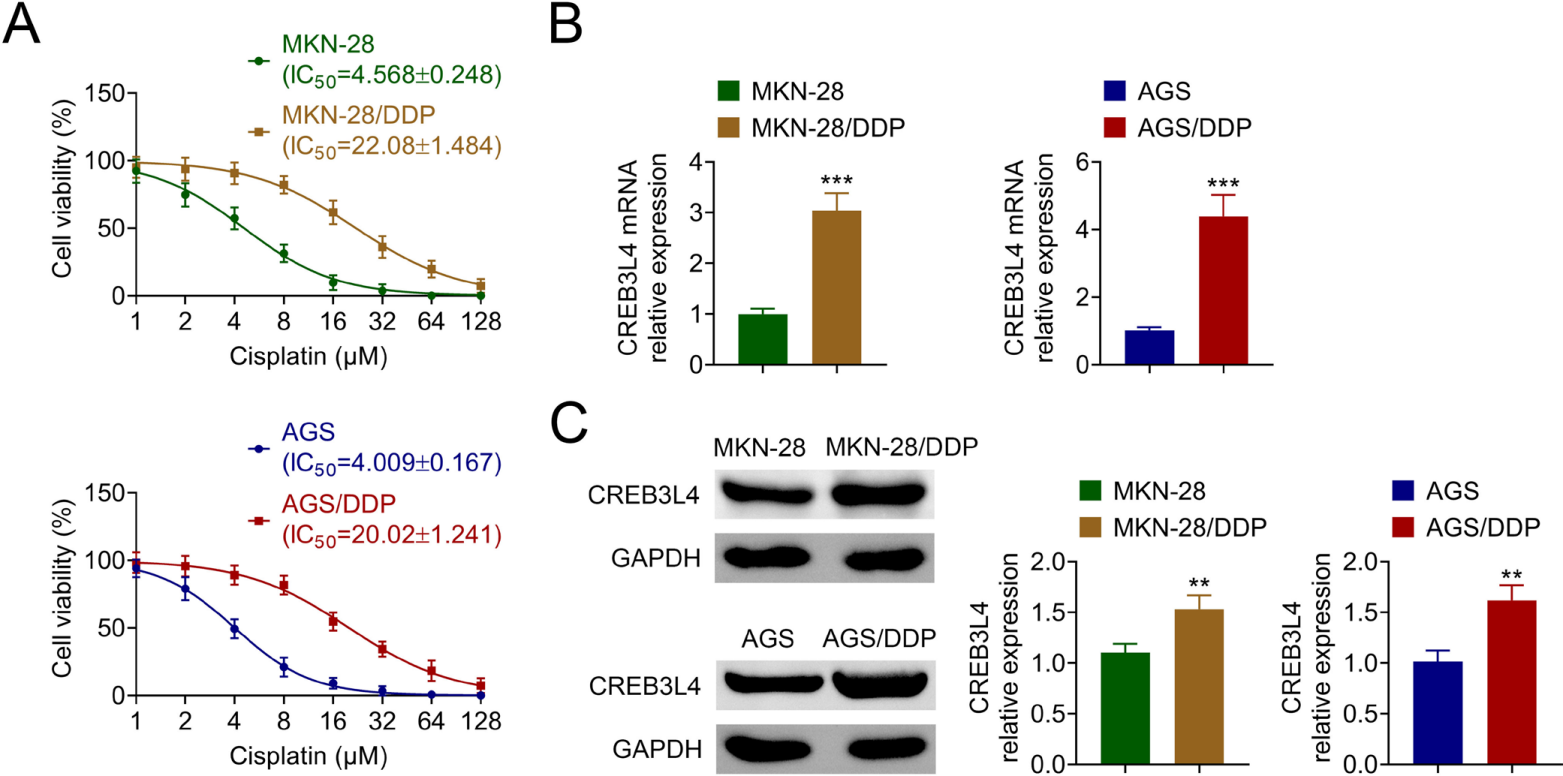
CREB3L4 is highly expressed in cisplatin‐resistant gastric cancer cells. (A) Dose‐dependent effects of cisplatin on cell viability in MKN‐28, AGS, MKN‐28/DDP, and AGS/DDP cells. IC_50_ values were calculated. (B) qPCR analysis showing the mRNA expression levels of CREB3L4 in the indicated groups. (C) Immunoblot analysis and corresponding densitometric quantification of CREB3L4 protein expression. Data are expressed as mean ± S.E.M. (*n* = 5 per group). **p < 0.01, ***p < 0.001. Each experiment was repeated three times.

First, we examined the viability of gastric cancer cell lines and calculated the IC_50_ to confirm the establishment of cisplatin‐resistant GC cells. As shown in Figure 1A, cell viability decreased in a dose‐dependent manner following cisplatin treatment. The IC_50_ values for cisplatin were significantly higher in MKN‐28/DDP (22.08 ± 1.484 μM) and AGS/DDP (20.02 ± 1.241 μM) cells than in their parental MKN‐28 (4.568 ± 0.248 μM) and AGS (4.009 ± 0.167 μM) cells (*p* < 0.001), confirming the successful establishment of cisplatin‐resistant sublines. We then assessed the expression of CREB3L4 in GC cells. As shown in Figure 1B, the mRNA expression level of CREB3L4 was significantly upregulated in MKN‐28/DDP (3.1‐fold, *p* < 0.01) and AGS/DDP (4.2‐fold, *p* < 0.001) cells compared with their parental counterparts. Consistently, immunoblot analysis showed that the protein expression level of CREB3L4 was also markedly increased in MKN‐28/DDP (1.55‐fold, *p* < 0.001) and AGS/DDP (1.60‐fold, *p* < 0.001) cells relative to parental cells (Figure 1C).

To further characterize the cisplatin‐resistant gastric cancer cell lines, we assessed the stability of cisplatin resistance after cisplatin withdrawal and compared the baseline growth rates between parental and resistant cells. As shown in Figure S1A, both MKN‐28/DDP and AGS/DDP cells exhibited markedly higher IC_50_ values than their parental counterparts, and this resistant phenotype remained largely stable after 4 weeks of culture in cisplatin‐free medium, although a slight reduction in IC_50_ was observed in the withdrawal sublines. In addition, baseline growth analysis showed that resistant and parental cells displayed similar proliferation trends on days 1 and 3, whereas the resistant cells showed slightly lower OD values by day 5 (Figure S1B), indicating that the acquisition of cisplatin resistance exerted only a modest effect on basal proliferative capacity. These results indicate that CREB3L4 is highly expressed in cisplatin‐resistant gastric cancer cells.

### 3.2 Knockdown of CREB3L4 Enhanced Cisplatin Sensitivity, Suppressed Cell Proliferation, and Stimulated Cell Apoptosis in Cisplatin‐Resistant Gastric Cancer Cells

**FIGURE 2:**
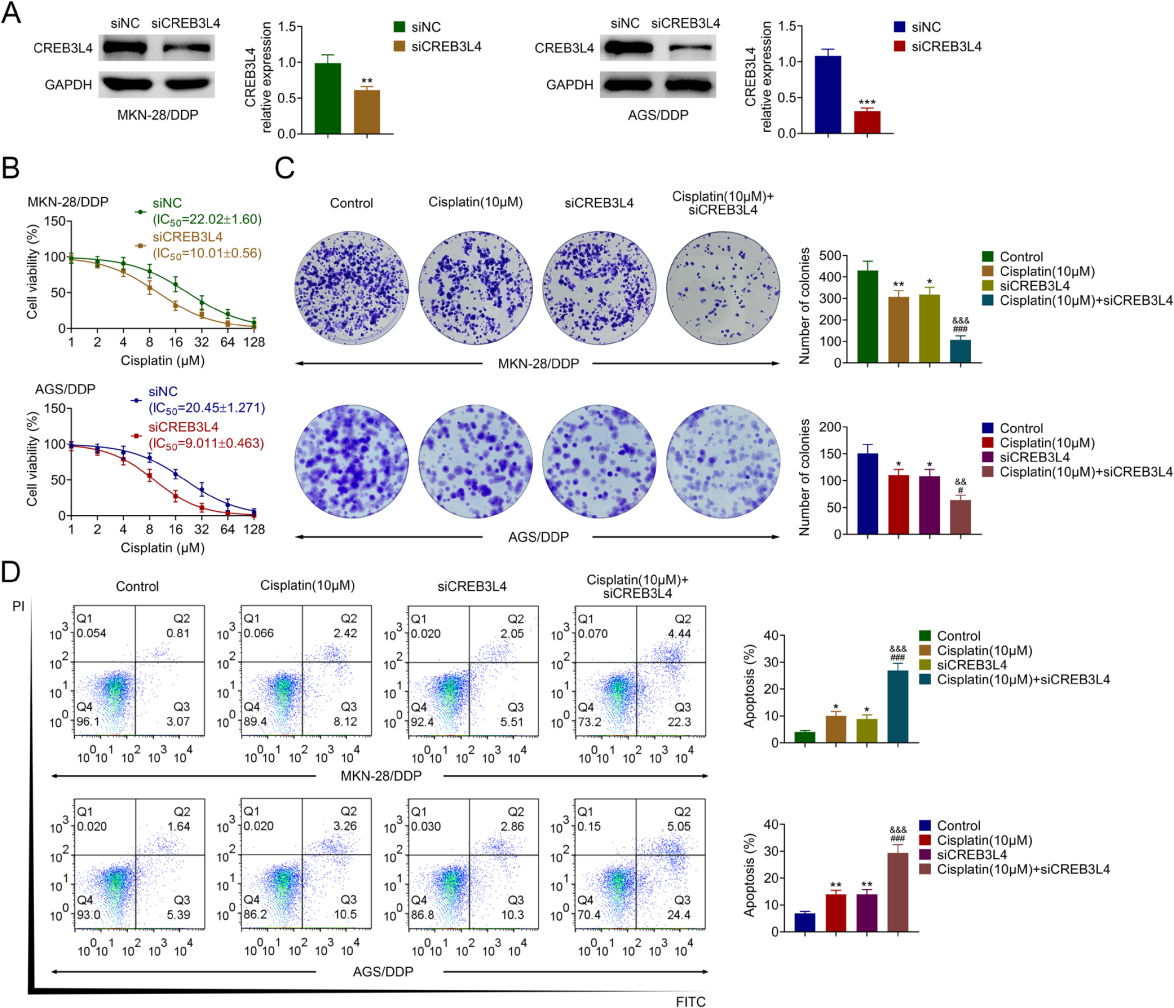
Knockdown of CREB3L4 enhances cisplatin sensitivity, suppresses cell proliferation, and promotes apoptosis in cisplatin‐resistant gastric cancer cells. (A) Immunoblot analysis of CREB3L4 expression in MKN‐28/DDP and AGS/DDP cells transfected with control siRNA (siNC) or CREB3L4 siRNA (siCREB3L4). GAPDH served as the loading control. Densitometric quantification is shown on the right. (B) Dose–response curves of cell viability in MKN‐28/DDP and AGS/DDP cells transfected with siNC or siCREB3L4 and treated with the indicated concentrations of cisplatin for 48 h. IC₅₀ values are shown in the legends. (C) Representative images of colony formation assays in MKN‐28/DDP and AGS/DDP cells under the indicated conditions. Quantification of colony formation rates is shown on the right. (D) Flow cytometry analysis of Annexin V‐FITC/PI‐stained cells under the indicated conditions. Quantification of apoptotic cells is shown on the right. Data are expressed as mean ± S.E.M. (*n* = 5 per group). *p < 0.05, **p < 0.01, ***p < 0.001 versus control, ^&^ *p* < 0.05, ^&&&^ *p* < 0.001 cisplatin (10 μM) + siCREB3L4 versus cisplatin (10 μM), ^##^ *p* < 0.01, ^###^ *p* < 0.001 cisplatin (10 μM) + siCREB3L4 versus siCREB3L4. Each experiment was repeated three times.

We further explored the effects of CREB3L4 on cisplatin‐resistant GC cells by transfecting si‐CREB3L4 to knock down CREB3L4 expression. The silencing efficiency of si‐CREB3L4 was confirmed by immunoblot analysis (Figure 2A). The results showed that knockdown of CREB3L4 significantly decreased the IC_50_ values of cisplatin in both MKN‐28/DDP and AGS/DDP cells (Figure 2B). In addition, the colony formation assay indicated that CREB3L4 knockdown reduced the colony‐forming ability of these cells both in the presence and absence of cisplatin treatment (Figure 2C). As shown in Figure 2D, knockdown of CREB3L4 also significantly promoted apoptosis in cisplatin‐resistant GC cells. Taken together, these findings indicate that depletion of CREB3L4 enhances cisplatin sensitivity, suppresses cell proliferation, and stimulates apoptosis in cisplatin‐resistant gastric cancer cells.

### 3.3 CREB3L4 Knockdown Suppressed the Autophagy of Cisplatin‐Resistant GC Cells

**FIGURE 3:**
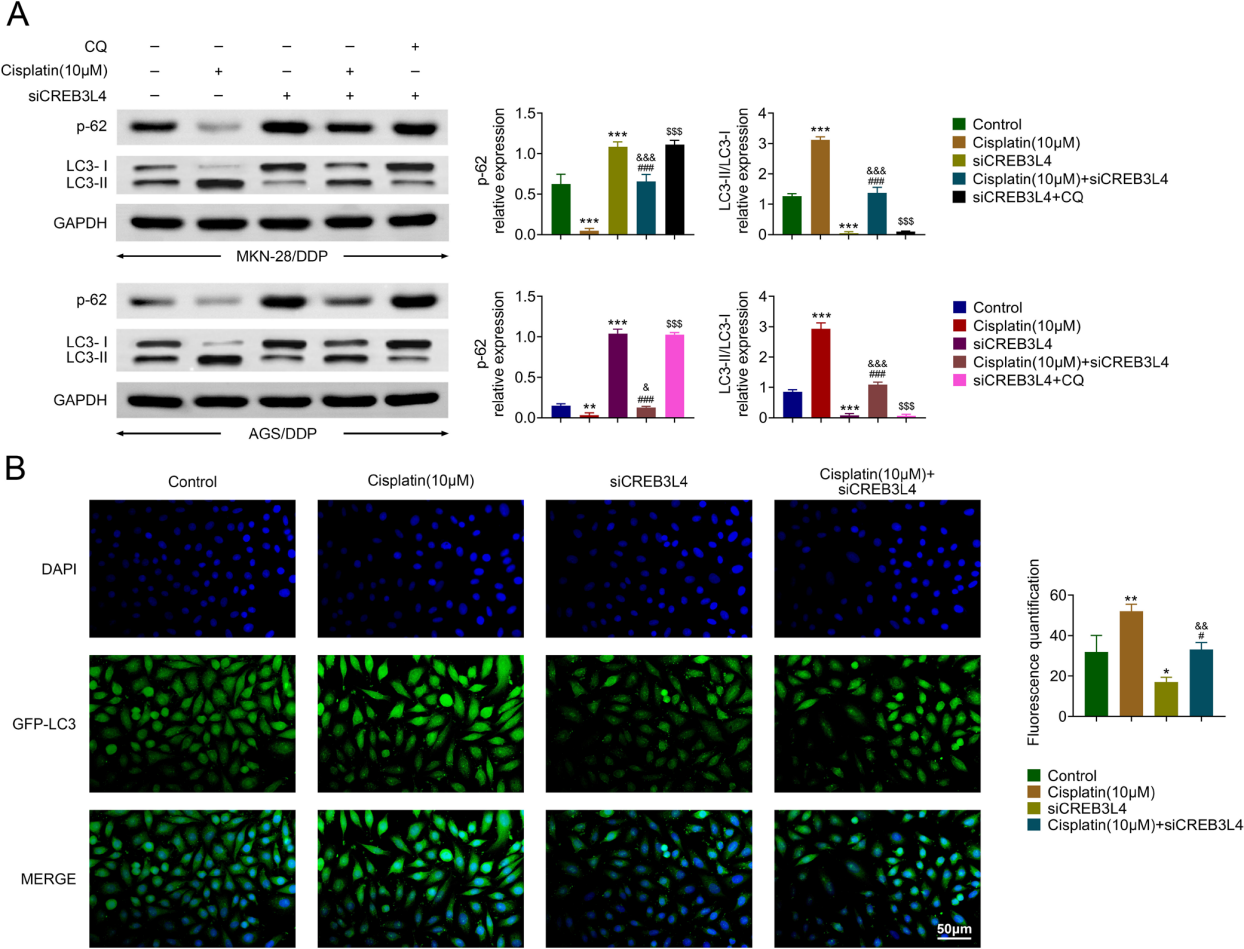
CREB3L4 knockdown suppresses autophagic flux in cisplatin‐resistant gastric cancer cells. (A) Western blot analysis of p62 and LC3‐I/II in MKN‐28/DDP and AGS/DDP cells under the indicated conditions: Control, cisplatin (10 μM), siCREB3L4, cisplatin (10 μM) + siCREB3L4, and siCREB3L4 + CQ (20 μM). GAPDH served as the loading control. Histograms on the right show densitometric quantification of p62 and LC3‐II/LC3‐I ratios. ***p < 0.001 versus control; ^&&&^ *p* < 0.001 for cisplatin (10 μM) + siCREB3L4 versus cisplatin (10 μM); ^$$$^ *p* < 0.001 for siCREB3L4 + CQ vs. siCREB3L4. (B) Representative fluorescence microscopy images of GFP‐LC3 puncta (green) and DAPI‐stained nuclei (blue) in MKN‐28/DDP cells under the indicated conditions. Scale bar = 50 μm. Quantification of GFP‐LC3 fluorescence intensity is shown on the right. **p < 0.01, ***p < 0.001 versus control; ^&&^ *p* < 0.01 for cisplatin (10 μM) + siCREB3L4 versus cisplatin (10 μM). Each experiment was repeated three times.

To further determine whether CREB3L4 regulates autophagic flux in cisplatin‐resistant gastric cancer cells, we performed LC3 turnover assays using chloroquine (CQ), a lysosomal inhibitor that blocks autophagosome degradation. Western blot analysis showed that CREB3L4 silencing markedly increased p62 accumulation and decreased the LC3‐II/LC3‐I ratio in both MKN‐28/DDP and AGS/DDP cells, indicating impaired autophagy. Upon CQ treatment, LC3‐II levels were further elevated, confirming that these changes reflected autophagic flux inhibition rather than a reduction in autophagosome formation (Figure 3A). Quantitative analysis of band intensities further demonstrated significant suppression of autophagy in the siCREB3L4 and siCREB3L4 + CQ groups compared with the cisplatin‐treated group (Figure 3A). Next, we assessed autophagy using a GFP‐LC3 reporter assay, which visualizes autophagosome formation through fluorescent puncta. CREB3L4 knockdown reduced GFP‐LC3 puncta intensity, further indicating suppression of autophagic activity (Figure 3B). Taken together, these results suggest that CREB3L4 knockdown suppresses autophagy in cisplatin‐resistant GC cells.

### 3.4 CREB3L4 Knockdown Suppressed the Expression of BAG3 in Cisplatin‐Resistant GC Cells

**FIGURE 4:**
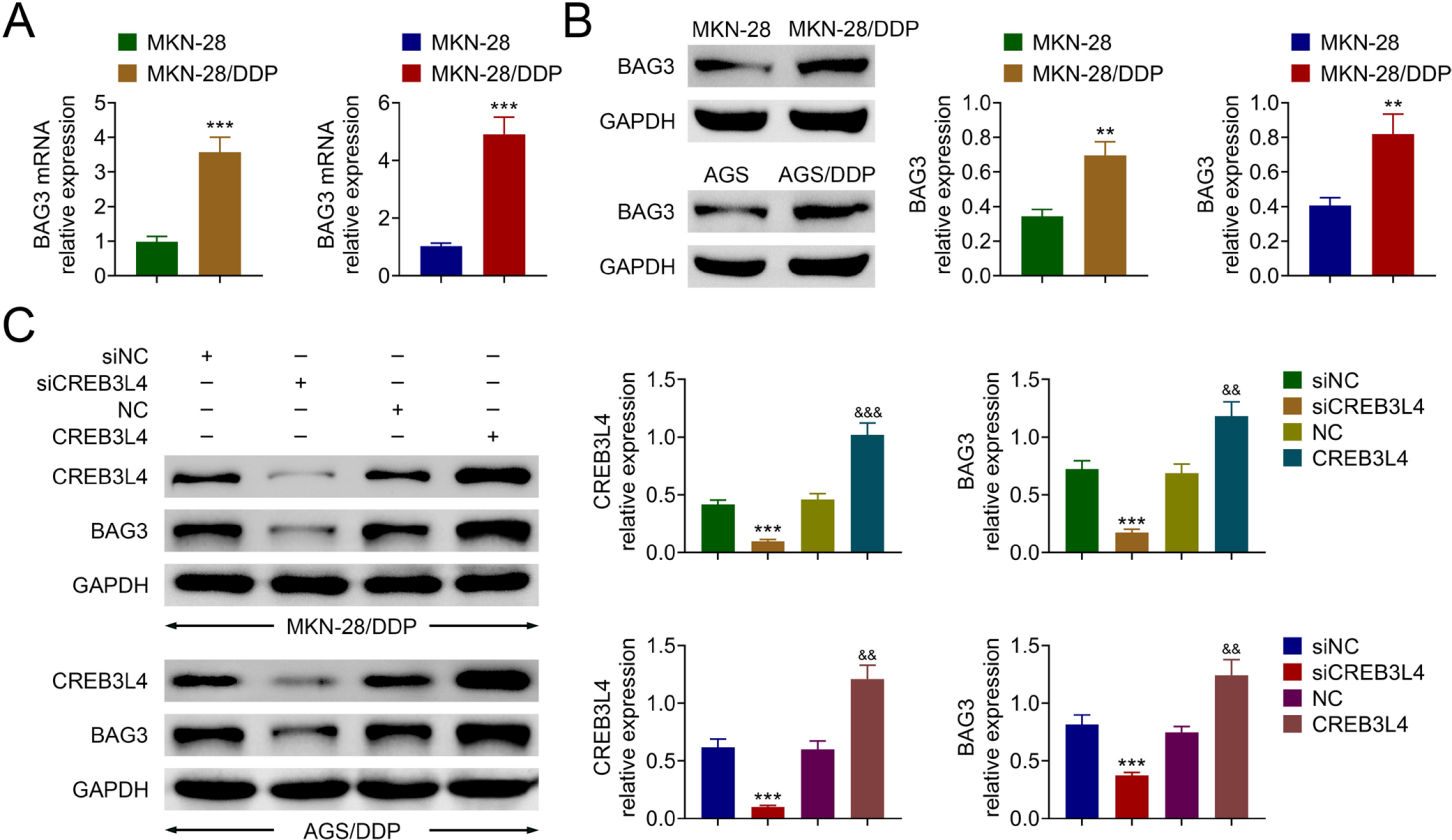
CREB3L4 knockdown suppresses BAG3 expression in cisplatin‐resistant GC cells. (A) qPCR analysis showing BAG3 expression in MKN‐28/DDP and AGS/DDP cells transfected with control siRNA or CREB3L4 siRNA. (B) Immunoblot analysis of BAG3 expression under the same conditions. (C) CREB3L4 and BAG3 expression levels in MKN‐28/DDP and AGS/DDP cells under the indicated treatments. Data are expressed as mean ± S.E.M. (*n* = 5 per group). **p < 0.01, ***p < 0.001 versus control, ^&&^ *p* < 0.01, ^&&&^ *p* < 0.001 cisplatin (10 μM) + siCREB3L4 versus cisplatin (10 μM). Each experiment was repeated three times.

Previous studies have shown that CREB3L4 can mediate the expression of BAG3, which may in turn influence the progression of GC. We therefore investigated the effects of CREB3L4 on BAG3 expression in both parental and cisplatin‐resistant GC cells. qPCR analysis showed that BAG3 was upregulated in cisplatin‐resistant GC cells (Figure 4A). Consistently, immunoblot analysis also demonstrated increased BAG3 protein expression in these cells (Figure 4B). Subsequently, we examined BAG3 expression after CREB3L4 overexpression or depletion. We found that depletion of CREB3L4 inhibited BAG3 expression, whereas overexpression of CREB3L4 promoted BAG3 expression (Figure 4C). Taken together, these findings indicate that CREB3L4 mediates BAG3 expression in cisplatin‐resistant GC cells.

### 3.5 Overexpression of BAG3 Rescues the Inhibitory Effects of CREB3L4 Knockdown on Autophagy and Cisplatin Resistance in Gastric Cancer Cells

**FIGURE 5:**
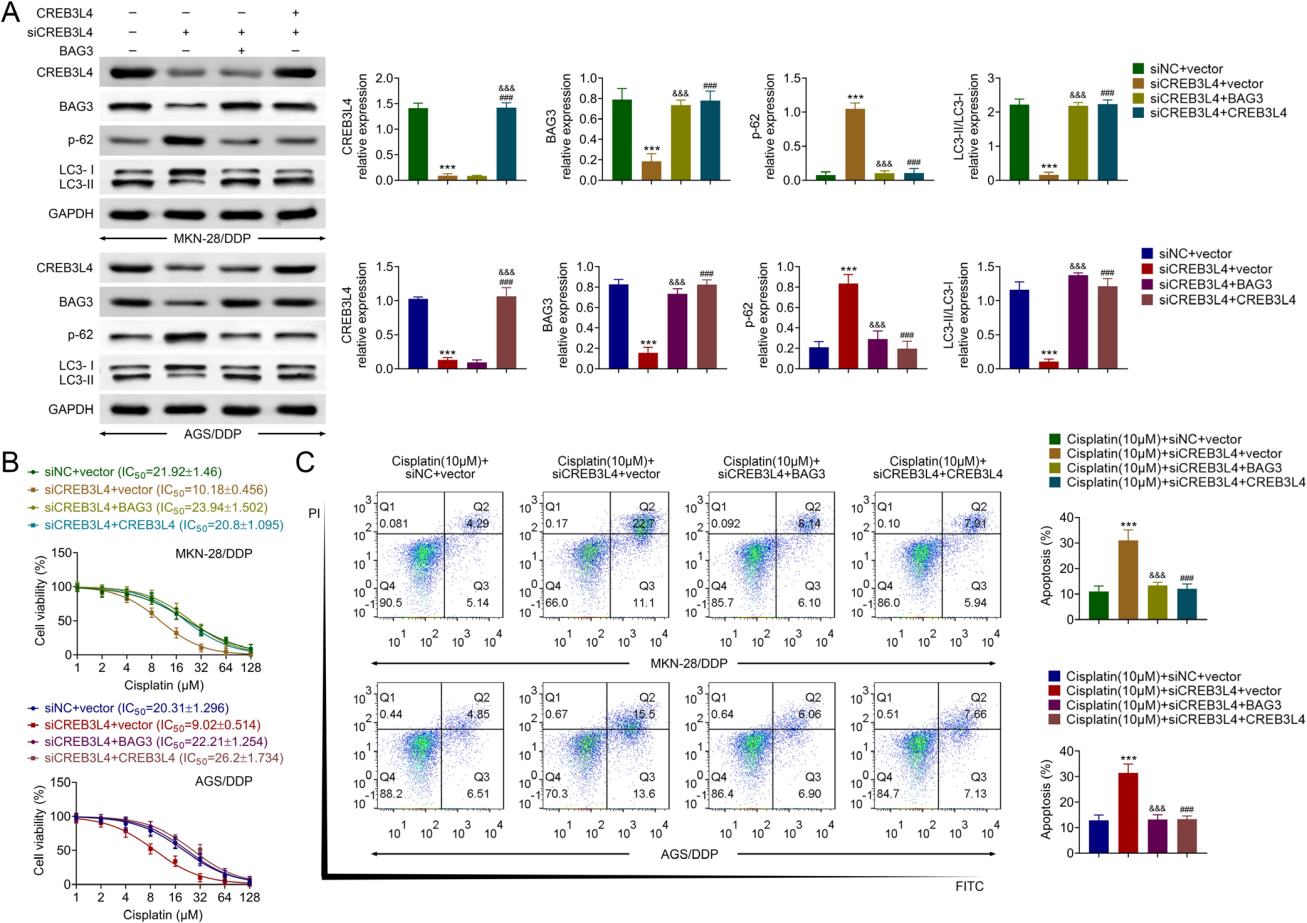
Overexpression of BAG3 rescues the inhibitory effects of CREB3L4 knockdown on autophagy and cisplatin resistance in gastric cancer cells. (A) Western blot analysis of CREB3L4, BAG3, p62, and LC3‐I/II in MKN‐28/DDP and AGS/DDP cells treated with siNC + vector, siCREB3L4 + vector, siCREB3L4 + BAG3, or siCREB3L4 + CREB3L4. GAPDH served as the loading control. Histograms show densitometric quantification of the indicated proteins. (B) Dose–response curves of cell viability in MKN‐28/DDP and AGS/DDP cells following cisplatin treatment (0–128 μM) under the indicated conditions. IC_50_ values are shown in the legends. (C) Flow cytometry analysis of Annexin V‐FITC/PI‐stained MKN‐28/DDP and AGS/DDP cells under the indicated conditions. Quantification of apoptotic cells is shown on the right. **p < 0.01, ***p < 0.001 versus control, ^&&^ *p* < 0.01, ^&&&^ *p* < 0.001 cisplatin (10 μM) + siCREB3L4 versus cisplatin (10 μM). Each experiment was repeated three times.

To further verify whether BAG3 mediates the effects of CREB3L4 on autophagy and cisplatin resistance, rescue experiments were performed by co‐transfecting siCREB3L4 with BAG3 or CREB3L4 overexpression plasmids in MKN‐28/DDP and AGS/DDP cells. Western blot analysis showed that CREB3L4 silencing significantly decreased BAG3 expression and the LC3‐II/LC3‐I ratio, while increasing p62 accumulation, indicating impaired autophagy. In contrast, overexpression of BAG3 or CREB3L4 reversed these changes, as evidenced by restoration of the LC3‐II/LC3‐I ratio and reduction of p62 expression (Figure 5A). Quantitative analysis further confirmed that overexpression of either BAG3 or CREB3L4 markedly restored the autophagic activity suppressed by siCREB3L4 (Figure 5A). Cell viability assays revealed that CREB3L4 knockdown substantially reduced the IC_50_ of cisplatin in both cell lines, whereas re‐expression of BAG3 or CREB3L4 restored cisplatin tolerance (Figure 5B). Moreover, flow cytometric analysis showed that CREB3L4 silencing significantly promoted apoptosis; while overexpression of BAG3 or CREB3L4 markedly attenuated the apoptosis induced by siCREB3L4 (Figure 5C). Taken together, these results indicate that CREB3L4 enhances cisplatin resistance in gastric cancer cells by upregulating BAG3‐dependent autophagy, thereby suppressing apoptosis.

## 4 Discussion

Cisplatin ranks second in comprehensive evaluations that consider efficacy, clinical applicability, and market utilization, and has often been referred to as the “cancer penicillin” because of its widespread use in oncology [18, 19]. In certain malignancies, such as testicular cancer and ovarian cancer, the initial cure rates reach approximately 100% and 85%, respectively [20]. Cisplatin also plays an important role in the chemotherapy of gastric cancer [21]. However, given the heterogeneity of tumors, particularly in gastric cancer, advanced‐stage disease is highly prone to the development of chemoresistance. Chemotherapy resistance in gastric cancer therefore represents a major clinical challenge, with cisplatin resistance being prominent [22]. To overcome this limitation, it remains essential to further elucidate the molecular mechanisms underlying cisplatin resistance and develop effective therapeutic strategies targeting these pathways, which is critical for improving patient prognosis. In the present study, we demonstrated that CREB3L4 is highly expressed in cisplatin‐resistant gastric cancer cells, and that knockdown of CREB3L4 inhibits autophagy and reduces cisplatin resistance in these cells.

Herein, we constructed cisplatin‐resistant GC cell lines and investigated the roles of CREB3L4 and BAG3 using immunoblotting and qPCR assays. Our results showed that BAG3 expression was inhibited following CREB3L4 knockdown in gastric cancer cells. Furthermore, based on immunoblot and immunofluorescence analyses, we found that the knockdown of CREB3L4 inhibited autophagy and alleviated cisplatin resistance in gastric cancer cells. In addition, flow cytometry and colony formation assays demonstrated that CREB3L4 depletion promoted apoptosis and inhibited cell proliferation through downregulation of BAG3. The role of CREB3L4 in tumor progression has been increasingly recognized across multiple cancer types [8, 23]. For instance, CREB3L4 has been reported to promote angiogenesis and tumor progression in gastric cancer via mediating VEGFA expression [23]. In addition, CREB3L4 has been shown to influence the viability of prostate cancer cells [8]. Collectively, these studies support the notion that CREB3L4 plays an important role in cancer progression.

Our findings also revealed that CREB3L4 knockdown suppressed the expression of BAG3 in cisplatin‐resistant GC cells, whereas overexpression of BAG3 reversed the effects of CREB3L4 depletion in these cells. BAG3 is a member of the BAG family and regulates several key markers of cancer. Mechanistically, BAG3 can interact with Bcl‐2 to inhibit apoptosis [14]. Previous studies have demonstrated that BAG3 is involved in drug resistance in breast and ovarian cancers through the regulation of autophagy [15, 16]. In addition, the role of BAG3 in bovine *Deltapapillomavirus*‐regulated autophagy has also been reported [24].

Similarly, our findings indicate that CREB3L4 regulates autophagy through modulation of BAG3 expression in cisplatin‐resistant GC cells. BAG3 has also been shown to induce fibroblasts to release key cytokines [25] and to induce α‐SMA expression in human fibroblasts [26]. Moreover, suppression of BAG3 has been reported to enhance the anticancer effects of shikonin in hepatocellular carcinoma [27]. Several studies have further confirmed the role of BAG3 in regulating cell viability, apoptosis, and autophagy in gastric cancer cells [28]. In line with these observations, our results demonstrate that CREB3L4 exerts its effects on gastric cancer progression, at least in part, via BAG3.

Our data demonstrated that CREB3L4 knockdown led to the accumulation of p62 and a reduction in the LC3‐II/LC3‐I ratio, which together indicate suppression of autophagic flux rather than activation. Consistent with the reduction in autophagy, apoptosis was markedly enhanced in CREB3L4‐depleted cells, as evidenced by increased Annexin V–positive cell populations and elevated levels of cleaved caspase‐3. These findings suggest that CREB3L4 promotes cisplatin resistance by maintaining a cytoprotective level of autophagy, whereas its depletion shifts the cellular balance toward apoptosis.

Despite the consistent in vitro evidence supporting the role of the CREB3L4/BAG3/autophagy axis in cisplatin resistance, several limitations of this study should be acknowledged. First, all experiments were conducted using established gastric cancer cell lines, and the findings lack in vivo validation. Although cell line models are valuable for mechanistic investigation, they cannot fully recapitulate the complexity of the tumor microenvironment, including interactions with stromal cells, immune components, and three‐dimensional architecture, all of which may influence drug resistance and autophagy regulation. Therefore, future studies employing xenograft models or patient‐derived organoids are warranted to further validate the therapeutic potential of targeting CREB3L4 in cisplatin‐resistant gastric cancer.

In summary, this study showed that CREB3L4 and BAG3 are overexpressed in cisplatin‐resistant GC cell lines, and that BAG3 expression is suppressed following CREB3L4 knockdown. Moreover, CREB3L4 depletion inhibits autophagy, promotes apoptosis, and suppresses cell proliferation through downregulation of BAG3.

## Funding

This work was supported by 2023 Nanchong Science and Technology plan project, 23YYJCYJ0145.

## Conflicts of Interest

The authors declare no conflicts of interest.

## Supporting information


**Figure S1:** Characterization of cisplatin‐resistant gastric cancer cells after cisplatin withdrawal and comparison of baseline growth rates. (A) Cisplatin sensitivity of parental, cisplatin‐resistant, and cisplatin‐withdrawal gastric cancer cells was evaluated by MTT assay after treatment with different concentrations of cisplatin for 48 h. (B) Baseline proliferation of parental and cisplatin‐resistant cells was assessed by MTT assay on days 1, 3, and 5 under normal culture conditions. **p < 0.01, ***p < 0.001 versus parental cells.

## Data Availability

All data generated or analyzed during this study are included in this published article. The datasets used and/or analyzed during the present study are available from the corresponding author on reasonable request.
