# Supplementary figures and images for: Knockdown of CREB3L4 Inhibits Autophagy and Reduces Cisplatin Resistance in Gastric Cancer Cells by Downregulating BAG3
Source: Kaohsiung J Med Sci. 2026 May 7:e70231. Online ahead of print. doi: 10.1002/kjm2.70231 (PMC13399867; doi:10.1002/kjm2.70231)

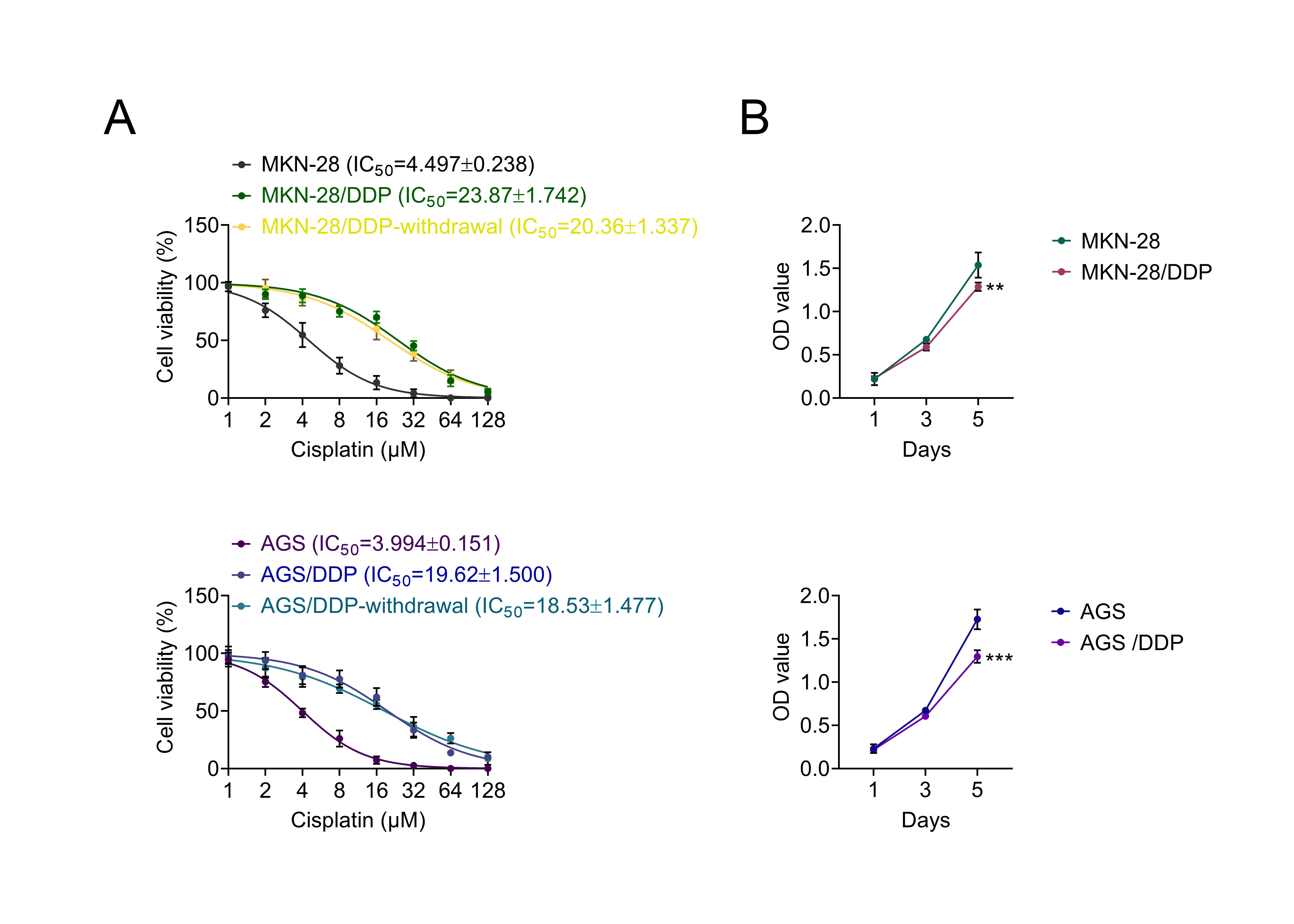

Supplement: Supplementary file 1 — Figure S1: Characterization of cisplatin‐resistant gastric cancer cells after cisplatin withdrawal and comparison of baseline growth rates. (A) Cisplatin sensitivity of parental, cisplatin‐resistant, and cisplatin‐withdrawal gastric cancer cells was evaluated by MTT assay after treatment with different concentrations of cisplatin for 48 h. (B) Baseline proliferation of parental and cisplatin‐resistant cells was assessed by MTT assay on days 1, 3, and 5 under normal culture conditions. **p < 0.01, ***p < 0.001 versus parental cells. [file KJM2-9999-e70231-s001.jpg]
